# Supplementary figures and images for: Natural diversity of the honey bee (Apis mellifera) gut bacteriome in various climatic and seasonal states
Source: PLoS One. 2022 Sep 9;17(9):e0273844. doi: 10.1371/journal.pone.0273844 (PMC9462563; doi:10.1371/journal.pone.0273844)

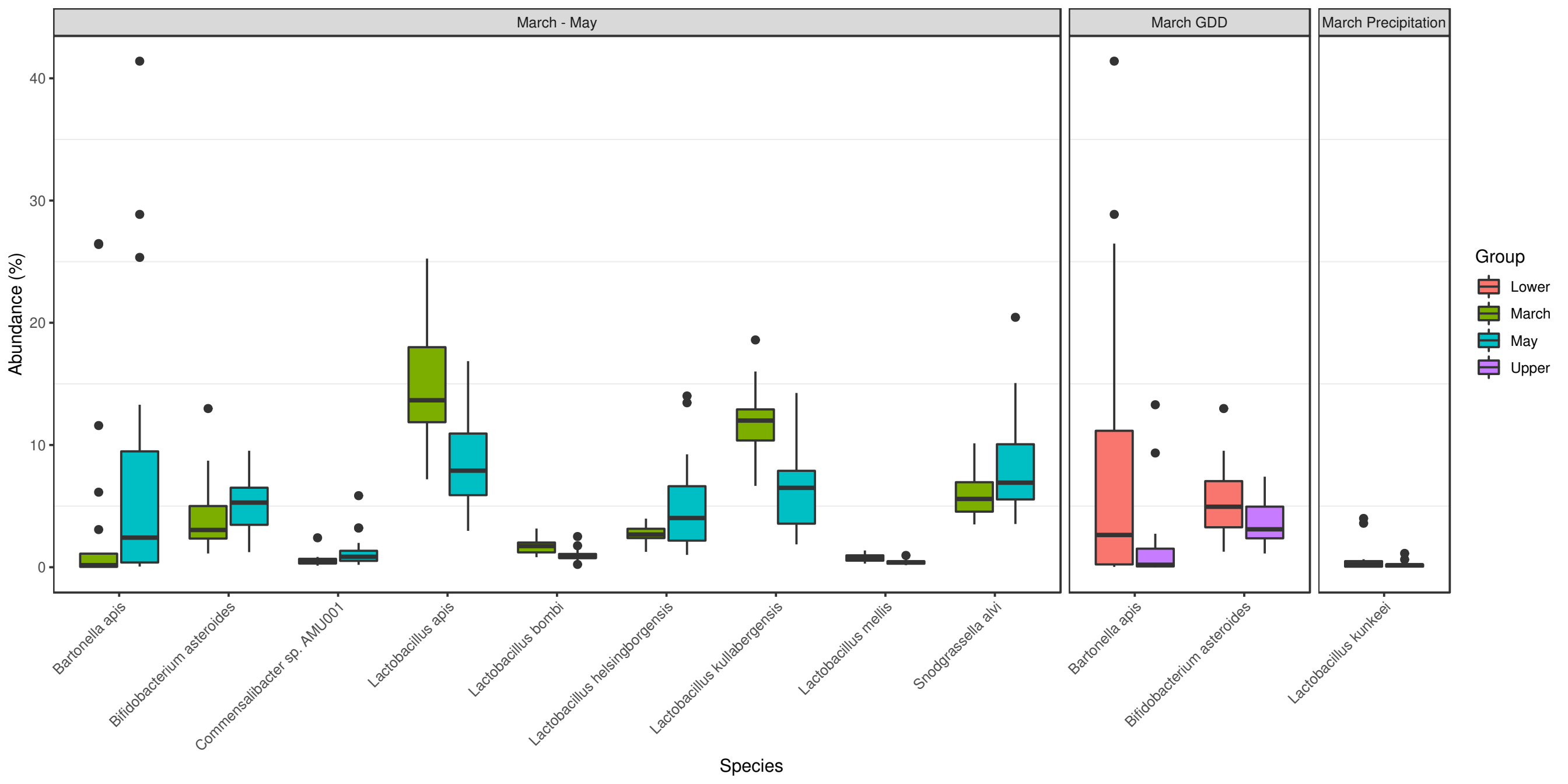

Supplement: S1 Fig — Boxplots denoting the actual point distribution for the differential species in comparisons were significant. (PDF) [file pone.0273844.s001.pdf]
