# Supplementary material for: Natural diversity of the honey bee (Apis mellifera) gut bacteriome in various climatic and seasonal states
Source: PLoS One. 2022 Sep 9;17(9):e0273844. doi: 10.1371/journal.pone.0273844 (PMC9462563; doi:10.1371/journal.pone.0273844)
Supplement: S1 Table — During the sampling in May, in two apiaries (ID: 6, 13) only two of the three colonies from the March sampling period were accessible so only these two were sampled. (PDF) [file pone.0273844.s002.pdf]

Table S1

Apiary ID	Family ID	March<br>Number of workers collected	Number of removed intestinal tracts	Number of pooled guts for sequencing	May<br>Number of workers collected	Number of remo-ved intestinal tracts	Number of pooled guts for sequencing
1	a	20	10	30	20	10	30
1	b	20	10		20	10	
1	c	20	10		20	10	
2	a	20	10	30	20	10	30
2	b	20	10		20	10	
2	c	20	10		20	10	
3	a	20	10	30	20	10	30
3	b	20	10		20	10	
3	c	20	10		20	10	
4	a	20	10	30	20	10	30
4	b	20	10		20	10	
4	c	20	10		20	10	
5	a	20	10	30	20	10	30
5	b	20	10		20	10	
5	c	20	10		20	10	
6	a	20	10	30	20	10	20
6	b	20	10		20	10	
6	c	20	10		0	0	
7	a	20	10	30	20	10	30
7	b	20	10		20	10	
7	c	20	10		20	10	
8	a	20	10	30	20	10	30
8	b	20	10		20	10	
8	c	20	10		20	10	
9	a	20	10	30	20	10	30
9	b	20	10		20	10	
9	c	20	10		20	10	
10	a	20	10	30	20	10	30
10	b	20	10		20	10	
10	c	20	10		20	10	
11	a	20	10	30	20	10	30
11	b	20	10		20	10	
11	c	20	10		20	10	
12	a	20	10	30	20	10	30
12	b	20	10		20	10	
12	c	20	10		20	10	
13	a	20	10	30	20	10	20
13	b	20	10		20	10	
13	c	20	10		0	0	
14	a	20	10	30	20	10	30
14	b	20	10		20	10	
14	c	20	10		20	10	
15	a	20	10	30	20	10	30
15	b	20	10		20	10	
15	c	20	10		20	10	
16	a	20	10	30	20	10	30
16	b	20	10		20	10	
16	c	20	10		20	10	
17	a	20	10	30	20	10	30
17	b	20	10		20	10	
17	c	20	10		20	10	
18	a	20	10	30	20	10	30
18	b	20	10		20	10	
18	c	20	10		20	10	
19	a	20	10	30	20	10	30
19	b	20	10		20	10	
19	c	20	10		20	10	
20	a	20	10	30	20	10	30
20	b	20	10		20	10	
20	c	20	10		20	10	
